# The Role of MET in Resistance to EGFR Inhibition in NSCLC: A Review of Mechanisms and Treatment Implications

**DOI:** 10.3390/cancers15112998

**Published:** 2023-05-31

**Authors:** Susan L. Feldt, Christine M. Bestvina

**Affiliations:** Department of Medicine, University of Chicago, Chicago, IL 60637, USA; sfeldt@uchicagomedicine.org

**Keywords:** NSCLC, *EGFR*, *mesenchymal-epithelial transition*, *MET* amplification, tyrosine kinase inhibitor, antibody drug conjugate

## Abstract

**Simple Summary:**

In non-small cell lung cancer that has spread to other locations in the body, identifying genetic abnormalities in a patient’s cancer has allowed for the development of targeted treatments. For cancers that have genetic changes in *epidermal growth factor receptor (EGFR)*, treatments such as osimertinib have allowed patients to live longer. However, these cancers do eventually continue to grow after targeted therapy. In this paper, we aimed to summarize the current research identifying changes in the *mesenchymal-epithelial transition (MET)* gene that occur after EGFR-targeted therapy, allowing the cancer to become resistant. We summarized current medications that target MET, and early findings from trials that used medications targeting both EGFR and MET together. Targeting both mechanisms at the same time could be a promising new treatment strategy, and larger trials studying these treatments in combination are currently ongoing to understand the potential benefit to patients.

**Abstract:**

Utilizing targeted therapy against activating mutations has opened a new era of treatment paradigms for patients with advanced non-small cell lung cancer (NSCLC). For patients with *epidermal growth factor (EGFR)*-mutated cancers, EGFR inhibitors, including the third-generation tyrosine kinase inhibitor (TKI) osimertinib, significantly prolong progression-free survival and overall survival, and are the current standard of care. However, progression after EGFR inhibition invariably occurs, and further study has helped elucidate mechanisms of resistance. Abnormalities in the *mesenchymal-epithelial transition (MET)* oncogenic pathway have been implicated as common alterations after progression, with *MET* amplification as one of the most frequent mechanisms. Multiple drugs with inhibitory activity against MET, including TKIs, antibodies, and antibody–drug conjugates, have been developed and studied in advanced NSCLC. Combining MET and EGFR is a promising treatment strategy for patients found to have a *MET*-driven resistance mechanism. Combination TKI therapy and EGFR-MET bispecific antibodies have shown promising anti-tumor activity in early clinical trials. Future study including ongoing large-scale trials of combination EGFR-MET inhibition will help clarify if targeting this mechanism behind EGFR resistance will have meaningful clinical benefit for patients with advanced EGFR-mutated NSCLC.

## 1. Introduction

Identifying activating mutations and the development of targeted therapies for these mutations have made precision medicine a reality for the care of patients with non-small cell lung cancer (NSCLC). In patients with advanced or metastatic NSCLC, molecular testing is recommended by the National Comprehensive Cancer Network (NCCN) guidelines for established molecular biomarkers [1]. An *epidermal growth factor receptor (EGFR)* mutation is a commonly found oncogenic driver in advanced NSCLC [2]. Exon 19 deletions and exon 21 L858R substitutions are the most common *EGFR*-activating mutations, accounting for 80–90% of *EGFR*-positive tumors [3]. For patients with activating *EGFR*-mutated NSCLC, targeted therapy with an EGFR tyrosine kinase inhibitor (TKI) is standard of care [2]. 

First- and second-generation EGFR TKIs including erlotinib, afatinib, geftinib, and dacomitinib have inhibitory effects on EGFR. However, a common pattern seen when utilizing targeted therapy is the development of resistance to the targeted agent through additional mutations or alterations. This was seen after treatment with first- and second-generation EGFR inhibitors; resistance commonly developed through the T790M *EGFR* mutation [4]. The third generation EGFR TKI osimertinib was developed and shown to be able to overcome the T790M resistance mechanism and was initially approved as second-line therapy. In the FLAURA trial, osimertinib demonstrated superior efficacy to first and second generation EGFR TKIs, and is now recommended as first-line therapy in advanced *EGFR*-positive NSCLC [5,6]. 

However, despite the shifting treatment paradigm to first-line osimertinib, resistance inevitably develops. Next-line therapy for those who progress after osimertinib has typically consisted of platinum-based chemotherapy. The study of resistance mechanisms to osimertinib has revealed additional molecular targets for therapy, with *mesenchymal-epithelial transition (MET)* oncogene alterations as the most common mechanism. In analyses of patients in the FLAURA trial, in *EGFR*-positive NSCLC treated with first-line osimertinib who progress, *MET* amplification can be seen in 15% of patients [7]. In an analysis of circulating-tumor DNA from plasma samples at baseline and at disease progression after first-line treatment with osimertinib similarly found *MET* amplification as the most frequent genetic mechanism of resistance found, identified in 16% [8]. Identifying specific mechanisms of resistance in an individual tumor after progression following first-line osimertinib treatment and adapting treatment to target to the mechanism could prolong survival [9]. This is not exclusive to *EGFR* mutant tumors; *MET* amplification is also being identified in as a driver to resistance to ALK, RET, and ROS-1 fusion TKI treatment [10].

*MET* encodes for a receptor tyrosine kinase which is activated by hepatocyte growth factor (HGF), and is found primarily in epithelial cells [11]. Downstream *MET* signaling activates RAS-MAPK, PI3K, and STAT3 pathways, leading to cell migration, invasion, proliferation, and cell survival [10,12]. Increased *MET* signaling has been seen in many types of cancers, including NSCLC, including gastric cancers, colorectal cancer, and papillary renal carcinomas [13]. *MET* abnormalities occur through *MET* exon skipping and amplification [14,15]. With the further implication of the role of *MET* in resistance to EGFR TKIs and the development of MET targeted therapies, this has become a promising area of clinical development in the treatment of advanced NSCLC [16].

## 2. Molecular Mechanisms of Acquired Resistance

Further study of the genomic changes of patients who’ve progressed after treatment with osimertinib have elucidated a diverse range of mechanisms of resistance. This includes EGFR-dependent mechanisms, alterations in *MET*, *RET*, *BRAF*, *KRAS*, and *PI3K*, as well as histologic transformation. Patients may have more than one coexisting molecular mechanism contributing to resistance [17]. While the overall landscape of osimertinib resistance is diverse, *MET* alterations, particularly *MET* amplification, have been seen as the most frequent mechanism.

*MET* dysregulation most commonly occurs through amplification or exon 14 skipping mutations. Exon 14 skipping mutations are caused by a point mutation or deletion that leads to loss of exon 14. Without exon 14 appropriately transcribed, the MET protein is more stable and less prone to degradation, thereby increasing MET signaling [18]. In a treatment-naïve population with comprehensive genomic profiling, *MET exon 14* skipping mutations are found in about 3% of patients with NSCLC [19]. 

*MET* amplification results in an increase in the number of copies of *MET*, either through focal amplification or chromosome 7 polysomy. While focal *MET* amplification has been associated with oncogenicity, amplification through chromosome 7 polysomy is typically not. Focal *MET* amplification is found in 1–6% of treatment-naïve NSCLC [20], and has demonstrated sensitivity to MET inhibitors such as capmatinib [21]. *MET* amplification is seen more frequently after first-line treatment with osimertinib than in a treatment-naïve population and is the most common mechanism of resistance to osimertinib identified [7]. 

## 3. Testing of MET Alterations

Currently, there is not a uniform practice for how to perform *MET* testing at either time of diagnosis or at time of progression on targeted therapy. *MET* alterations can be tested for using a variety of methods, including both tumor tissue testing and liquid biopsy testing. *MET* amplification is traditionally diagnosed through fluorescence-in-situ-hybridization (FISH) of tissue obtained by biopsy. *MET* amplification by FISH is defined using two main strategies. The first determines gene copy number (GCN), with multiple cutoffs used, commonly a GCN of five or more, but also six or fifteen have been used as cutoffs. An alternative method is to control for chromosome 7 by using a ratio of *MET* per cell to chromosome 7 centromere (MET/CEP7). With this method, a *MET* to CEP7 ratio of greater than 2 is the typical definition of *MET* amplification [13].

NGS can also be used for the detection of *MET* amplification. As with FISH, there is not a consensus of copy number to define amplification. Unlike FISH, NGS does not control for chromosome 7 copy number [13]. There has been some discrepancy between *MET* amplification diagnosed with FISH compared to NGS, so a higher GCN cutoff of at least 10 is often used. With a higher cutoff, concordance with FISH is improved, but low to moderate *MET* amplification is less likely to be detected. At these levels, FISH has improved detection compared to NGS [22]. 

However, for detecting *MET exon 14* skipping mutations, NGS is overall the preferred strategy [23]. There is a suggestion that RNA-based NGS may be preferred to DNA-based NGS for in this setting, possibly due to heterogeneity in mutation variants resulting in exon 14 skipping [24]. Qt-PCR of *MET exon 14* skipping as a single gene testing could also be a reasonable approach [23]. 

IHC has also been studied for diagnosis of *MET* alterations. Different IHC cutoffs for determining high *MET* expression have been used in clinical trials and vary in both the minimum percent of tumor cells expressing *MET* and in level of IHC positivity (2+ vs. 3+). IHC appears to be less sensitive in detecting both exon 14 skipping mutations and *MET* amplification [25]. Thus, FISH and NGS are the preferred methods for detecting *MET* amplification. 

The use of cell-free DNA (cfDNA) testing in non-small cell lung cancer has increased dramatically over the past five years. Liquid biopsy can provide either a complementary or an alternative method of genomic profiling in addition to or in place of tissue moleculars. cfDNA NGS can also be used to diagnose alterations of *MET*, though interpretation of *MET* amplification can be difficult using this platform [26]. An additional benefit of this method is that cfDNA does not have the same heterogeneity and potential sampling error of a solid tissue biopsy. However, tumors may not shed enough genetic material for alterations to be detected, and this may lead to missing alterations [13]. Cell-free DNA could have a role as a molecular marker of response to treatment. In the VISION trial of tepotinib in advanced NSCLC with *MET exon 14* skipping mutations, cfDNA was obtained baseline and on treatment. A total of 67% of patients had a molecular response, which had a high concordance with clinical response [27]. 

Without a consensus in method of detecting *MET* alterations, clinical trials use multiple of these methods to determine what is considered *MET*-altered NSCLC, and future study could benefit from a standardized approach. However, given the implication of *MET* in resistance mechanisms, rebiopsy or liquid biopsy for genomic profiling of resistance is warranted in patients who progress after EGFR inhibition and may reveal *MET* alterations for targeted treatment.

## 4. MET Inhibition

### 4.1. Non-Selective MET TKIs 

TKIs with activity against MET are defined by how they bind MET as well as whether they are selective or non-selective for MET. Type 1 MET TKIs compete with ATP and are divided into Type 1a and Type 1b based on where they bind to MET. Type 1a binds the solvent front residue, which is not specific to MET, and are therefore non-selective MET inhibitors. Type 1b selectively binds MET. Type 2 MET TKIs differ in binding inactive MET and are also non-selective. Non-selective MET inhibitors include both crizotinib and cabozantinib. Crizotinib is a type 1a or non-selective MET TKI in that it inhibits MET in addition to ALK, ROS, and RON. It was initially approved for *ALK* or *ROS1* rearrangements in NSCLC [28,29]. In the phase I PROFILE 1001 trial, crizotinib was studied in advanced NSCLC with multiple other genetic alterations considering its non-specific inhibition. In 69 patients with *MET exon 14* alterations who received crizotinib, the objective response rate (ORR) was 32% (95% CI 21–45%) with a median progression free survival (PFS) of 7.3 months (95% CI 5.4–9.1 months). The most common adverse events seen with crizotinib included edema (51%), vision disorder (45%), diarrhea (39%) and vomiting (29%). Most common grade 3 or greater adverse events were transaminitis (4%) and dyspnea (4%) [30]. 

Cabozantinib is a type II non-selective MET TKI. In addition to MET activity, it also targets VEGFR, RET, TIE2, FLT-3, and KIT. Cabozantinib is currently approved for renal cell carcinoma, medullary thyroid carcinoma, and hepatocellular carcinoma. Studies of cabozantinib in NSCLC are more limited. In advanced NSCLC, cabozantinib has been studied in *EGFR* wildtype, but not mutant, patients. In a phase II trial, 42 patients with *EGFR* wildtype advanced non-squamous NSCLC who had received one or two previous treatments were randomized to receive erlotinib, cabozantinib, or combination erlotinib and cabozantinib. *MET* alterations were not specifically tested for. PFS was longer with cabozantinib alone (4.3 months, 95% CI 3.6–7.4) and with combination erlotinib and cabozantinib (4.7 months, 95% CI 2.4–7.4) than with erlotinib monotherapy (1.8 months; 95% CI 1.7–2.2 months). Adverse events with cabozantinib were most commonly fatigue (55%), diarrhea (50%), and nausea (45%). Of grade 3 or greater adverse events, hypertension (25%), fatigue (15%), and oral mucositis (10%) were most common [31].

### 4.2. Selective MET TKIs

Type 1b MET inhibitors are selective in their inhibition of MET. This class of drugs has had recent FDA approvals after demonstrating success in multiple recent clinical trials, based on an improvement in both efficacy as well as decreased toxicity given there is less off-target activity. This class includes tepotinib, capmatinib, and savolitinib. 

Tepotinib is a selective MET inhibitor that is FDA approved in patients with NSCLC with *MET exon 14* skipping alterations. The phase II VISION trial studied tepotinib in cohorts of both *MET* amplified and *MET exon 14* skipping as a mix of first-, second-, and third-line therapy. *MET exon 14* skipping was determined by liquid biopsy or RNA NGS of tissue. In 152 patients treated with tepotinib, the ORR was 46% (95% CI 36–61%) and was similar when stratified by prior therapy, and a median PFS of 8.5 months (95% CI 6.7–11.0) was seen. Grade 3 or greater adverse events occurred in 28% of patients, most commonly peripheral edema (7%), increased lipase (3%) and amylase (2%) [27]. 

The VISION cohort of *MET* amplification consisted of 24 patients with amplification determined by liquid biopsy. Median PFS was 4.2 months (95% CI 1.4-NE) in this cohort. The overall ORR was 42% (95% CI 22–63), with a 71% (95% CI 29–96) response rate seen in treatment-naïve patients, 30% (95% CI 7–65) as second-line therapy, and 29% (95% CI 4–71) as third-line therapy. A total of 67% of patients had treatment-related adverse events, most commonly peripheral edema (38%), generalized edema (17%), and constipation (17%). Grade 3 or greater adverse events were experienced by 29% of patients, most commonly peripheral edema (8%) and generalized edema (8%) [32]. 

Capmatinib is a selective MET inhibitor that is FDA approved for NSCLC with *MET exon 14* skipping mutations. In the GEOMETRY mono-1 phase II clinical trial, patients with *MET* dysregulated advanced NSCLC were treated with capmatinib. In those with *MET exon 14* skipping mutations, the ORR was 41% (95% CI 29–53) with a median PFS of 5.4 months (95% CI 4.2–7.0). In patients who had not received prior treatment, the ORR was higher at 68% (95% CI 48–84) with a higher median PFS as well at 12.4 months (95% CI 8.2-NE). However, in patients with *MET* amplification, efficacy was limited to those with high gene copy numbers, with an ORR of 29% (95% CI 19–41) and a median PFS of 4.1 months (95% CI 2.9–4.8) in those with a gene copy number of greater than 10. Peripheral edema (51% of patients) and nausea (45%) were the most common adverse events. Grade 3 or higher adverse events were seen in 67% of patients; most commonly peripheral edema (9%) and dyspnea (7%) [21]. 

Savolitinib is a selective MET TKI that is approved in China for NSCLC with *MET exon 14* skipping mutations. Savolitinib was studied in a phase II trial in China in advanced or metastatic NSCLC with an exon 14 skipping mutation and that has progressed or had toxicity to a prior treatment. The ORR was 49.2% (95% CI 31.1–55.3) with a PFS of 6.9 months (95% CI 4.6–8.3). All patients had a treatment-related adverse event, most commonly peripheral edema (54%), nausea (46%), and transaminitis (37–39%). Grade 3 or greater treatment-related adverse events were seen in 46% of patients, most commonly transaminitis (10–13%) and peripheral edema (9%) [33].

### 4.3. MET Antibodies

MET antibody-directed therapy is another area of active development and clinical investigation. One of the first promising signals of efficacy was emibetuzumab, a humanized immunoglobulin G4 monoclonal bivalent MET specific monoclonal antibody. Emibetuzumab has both ligand dependent and independent effects, both blocking HGF from binding MET and leading to MET being internalized and degraded. In a phase II trial, patients with stage IV EGFR mutated NSCLC were randomized after 8 weeks of erlotinib to continuing erlotinib monotherapy or to erlotinib plus emibetuzumab 750 mg infusion every 2 weeks. There was no difference in progression free survival between the overall groups (9.3 months with combination therapy and 9.5 months with erlotinib monotherapy). A post-hoc analysis of those with high *MET* expression was performed, defined as IHC with *MET* expression level of 3+ in at least 90% of tumor cells, which 24 patients met. With this definition of high *MET* expression, progression free survival with emibetuzumab plus erlotinib was 20.7 months vs. 5.4 months with erlotinib alone (HR = 0.39, 90% CI 0.17–0.91). Toxicity was higher in the combination arm, with peripheral edema (11.3% vs. 0%) and mucositis (15.5% vs. 8.6%) occurring more frequently than in those who received erlotinib alone [34]. 

Onartuzumab is a recombinant, humanized monoclonal antibody against *MET*. Onartuzumab blocks interaction with HGF by binding to extracellular MET. Phase II and III trials have studied onartuzumab in combination with erlotinib and chemotherapy without demonstrating a benefit in PFS or overall survival (OS) [35,36,37]. The phase III *MET* Lung trial studied onartuzumab with erlotinib or placebo infusion plus erlotinib. 499 patients with locally advanced or metastatic NSCLC with *MET* positive status defined as IHC 2+ or greater in at least 50% of cells who had progressed after platinum-based chemotherapy were enrolled. Shorter OS was seen in in the onartuzumab arm at 6.8 months vs. 9.1 months with placebo (HR = 1.27, 95% CI 0.98–1.65). Peripheral edema (21.8% vs. 7.8%) and hypoalbuminemia (17.3% vs. 3.7%) were more common in the onartuzumab arm than erlotinib monotherapy [38]. Grade 4 (5.2% vs. 2.9%) and grade 5 (6.9% and 4.1%) adverse events were more common in the onartuzumab group.

### 4.4. MET Antibody-Drug Conjugates

Teliso-V is an antibody-drug conjugate of the telisotuzumab humanized monoclonal antibody which targets c-MET conjugated to the microtubule inhibitor monomethyl auristatin E (MMAE). In a phase I/Ib study, patients with NSCLC and a c-MET H-score of at least 150 or local lab reported *MET* amplification or exon 14 skipping mutation received Teliso-V monotherapy. 40 c-MET+ patients were enrolled, and the ORR was 23% (95% CI 10.8–38.5) with median PFS of 5.2 months. A total of 65% of patients had grade 3 or greater adverse events, most commonly anemia (10%), fatigue (8%), and peripheral neuropathy (6%) [39]. Another MET-targeting antibody-drug conjugate (REGN5093-M114) is being studied in pre-clinical models and has shown promising anti-tumor activity in cells after progression following treatment with osimertinib and savolitinib [40].

## 5. Combination MET and EGFR Inhibitors

Considering the demonstrated benefit of EGFR inhibition in *EGFR*-mutated advanced NSCLC and the prevalence of *MET* dysregulation in resistance to third generation EGFR inhibitors, inhibition of EGFR and MET in combination has been a preferred strategy in clinical investigation in patients previously treated and progressed after initial EGFR inhibitor therapy. With uncertainty in how to treat patients with *MET*-amplification-mediated resistance to EGFR-TKI, real-world data does support the approach of combination EGFR and MET inhibition. 70 patients received either EGFR-TKI and crizotinib (n = 38), crizotinib monotherapy (n = 10), or chemotherapy (n = 22). PFS was longer in combination inhibition than with crizotinib monotherapy or chemotherapy (5.0 months vs. 2.3 and 2.9 months, respectively) [41]. More recently, combination EGFR/MET inhibition has been moved into studies in the first line.

### 5.1. EGFR TKI and MET TKI

With multiple EGFR and MET inhibitors available, trials have studied different combinations of these inhibitors to identify a dual-inhibition strategy with significant anti-tumor activity as well as an acceptable risk profile. Results of trials utilizing and EGFR TKI and a MET TKI are summarized in Table 1. 

In a study of 104 treatment-naïve patients with de novo *EGFR* positive and *MET* over-expressed advanced NSCLC, EGFR TKI monotherapy (n = 48), EGFR TKI plus crizotinib (n = 9), EGFR TKI plus chemotherapy (n = 12), and chemotherapy alone (n = 35) were compared. *MET* overexpression was defined by IHC with above-median H-score and 2+ or 3+ staining in 50% or greater of tumor cells. Notably, EGFR TKIs varied and included geftinib, erlotinib, afatinib, and osimertinib. This study showed that those treated with an EGFR-TKI (monotherapy and combination with crizotinib) had a longer PFS than chemotherapy (8.0 months vs. 4.0 months, HR = 0.50, 95% CI 0.21–0.80). EGFR TKI monotherapy or with crizotinib had comparable PFS (8.0 months vs. 8.5 months, HR = 0.96, 95% CI 0.44–2.09). Grade 3 or greater rashes were more common in patients who received EGFR-TKI plus crizotinib (0% vs. 33.3%) [42].

In a phase 1b/II trial, patients with *EGFR*-positive, *MET*-amplified NSCLC who had progressed on prior EGFR TKI treatment received capmatinib in combination with gefitinib. *MET* amplification was defined as GCN of at least 5 and/or a *MET*/centromere ratio of 2 or greater or MET overexpression with at least 50% of tumor cells with moderate or strong IHC staining. The ORR across the phase Ib/II study was 27%. In patients with high *MET* amplification defined as gene copy number of at least 6 (36 of the total 100 patients), a higher ORR of 47% was seen. The most common adverse events were nausea (28%), peripheral edema (22%), decreased appetite (21%), and rash (20%). Grade 3 or greater adverse events were seen in 33% of patients, most commonly increased amylase or lipase (both in 6%) [48]. 

TATTON was a phase 1b study of locally advanced or metastatic *MET*-amplified, *EGFR*-mutated NSCLC who had progressed on EGFR TKIs. Osimertinib was studied in combination with multiple other targeted therapies: selumetinib (MEK1/2 inhibitor), durvalumab (anti-PD-L1 monoclonal antibody), and savolitinib. *MET* amplification was defined as FISH with GCN of 5 of greater or *MET*-CEP7 ratio 2 or greater, IHC with 3+ expression in 50% of cells, or NGS with 5 or greater copies in 20% of tumor cells. Cohorts were stratified based on exposure to prior third generation EGFR TKI, and in those without prior third generation TKI, whether *EGFR T790M* was present [49].

In patients previously treated with a third generation EGFR TKI, the ORR of savolitinib plus osimertinib was 33% (95% CI 22–46). However, in those who had not been previously treated with a third generation EGFR TKI, ORR was even higher, ranging from 62–67% and regardless of T790M status [43]. Nausea (67%), rash (56%), and vomiting (50%) were the most common adverse events [50]. 

The combination of osimertinib and savolitinib was also studied in *EGFR* mutant NSCLC previously treated with first-line osimertinib with *MET* alterations in the ORCHARD study. *MET* amplification and exon 14 skipping mutations were included and identified by NGS of tumor biopsy. In 20 patients, an ORR of 41% was seen. A total of 30% had a grade 3 or greater adverse event, most commonly pneumonia and decreased neutrophil count (10% each) [51]. 

The phase II SAVANNAH trial is currently investigating savolitinib plus osimertinib as second-line therapy in patients with acquired resistance to osimertinib *MET* overexpression or amplification [44]. The phase II FLOWERS trial is ongoing and studying osimertinib with or without savolitinib as first-line therapy in patients with *MET*-amplified or overexpressed and *EGFR*-positive locally advanced or metastatic NSCLC [45].

In a phase 1b trial, combination savolitinib and gefitinib were studied in patients with *EGFR*-mutant *MET*-amplified advanced NSCLC in China after progression following prior EGFR TKI therapy. *MET* amplification was determined by FISH with a GCN or 5 or greater or *MET*-CEP7 of 2:1 or greater. ORR overall was 31%, with higher ORR seen in *EGFR T790M* negative (52%) and lower seen in *EGFR T790M* positive (9%). Most common adverse events were vomiting (46%), nausea (40%), and increased aspartate aminotransferase (39%). Most common grade 3 or greater adverse events included transaminitis (AST and ALT 7% each), and increased gamma-glutamyltransferase (5%) [46].

In a phase I/II dose escalation trial, combination capmatinib and erlotinib were studied in *MET*-positive NSCLC. This included a cohort of patients with *EGFR* mutations designated Cohort A with 12 patients. ORR in this cohort was 50%. Most common adverse events were rash (63%), fatigue (51%), and nausea (45.7%). Grade 3 or greater adverse events were seen in 34% of patients, most commonly decreased lymphocytes (9%), limb edema (6%), anorexia (6%), and increased lipase (6%) [52].

The INSIGHT-1 phase 1b/2 trial studied tepotinib plus gefitinib in patients with *EGFR*-mutated NSCLC that were T790M negative and *MET* overexpression (IHC 2+ or 3+) or *MET* amplified by FISH (GCN 5 or greater or *MET*/CEP 2 or greater). In the phase 2 trial, patients were randomly assigned to tepotinib plus gefitinib at the phase 1b determined dose of 500 mg or standard platinum doublet chemotherapy. Final analyses of phase 2 with 55 patients are now published, and in the group at large, median PFS was similar at 4.9 months with tepotinib and gefitinib vs. 4.4 months with chemotherapy. In the 19 patients with *MET* amplification, tepotinib plus geftinib had longer PFS (HR 0.13, 90% CI 0.04–0.43) and OS (HR 0.10, 90% CI 0.02–0.36) than chemotherapy. ORR was 66.7% with combination TKI therapy compared to 42.9% with chemotherapy [53]. Rates of treatment-related grade 3 or worse adverse events were similar between the groups (19% in the tepotinib/gefitinib group vs. 30% in chemotherapy), with increased amylase (16%) or lipase (13%) being the most common of grade 3 or greater adverse events with combination tepotinib and gefitinib [47]. 

INSIGHT 2 is an ongoing phase II trial of tepotinib plus osimertinib in advanced *EGFR*-mutant NSCLC with acquired resistance to first-line osimertinib and with *MET* amplification determined by FISH with GCN of 5 or greater or *MET*/CEP7 of 2 or greater [54]. Initial results from INSIGHT 2 were presented at ESMO 2022, and in 22 patients with at least 9 months follow up, an ORR of 54.5% was seen, as well as a similar safety profile. Primary analysis is planned for when all patients have had at least 9 months follow up [55].

### 5.2. EGFR TKI and MET Antibody

Results of combination EGFR- and MET-inhibition-utilizing antibodies and antibody drug conjugates are summarized in Table 2.

In a randomized phase II trial, patients with stage IV NSCLC with acquired resistance to erlotinib with increased *MET* expression were randomized 3:1 to either emibetuzumab with erlotinib or emibetuzumab monotherapy. Increased *MET* expression was defined IHC with as at least 10% of cells expressing MET at 2+. ORRs were low: 3% for emibetuzumab plus erlotinib (95% CI 0.4–10.5) and 4.3% for emibetuzumab monotherapy (95% CI 0.1–21.9). With combination therapy, fatigue (29%), diarrhea (25%), and nausea (23%) were the most common adverse events overall. Grade 3 or greater adverse events were seen in 24.1% of patients on combination therapy, most commonly dermatitis acneiform (6%) and hypoalbuminemia (3.6%) [56].

### 5.3. EGFR TKI and MET ADC

Teliso-V was studied in a phase 1b trial in combination with erlotinib in 42 patients with *EGFR* positive NSCLC who had progressed on a prior EGFR TKI. Patients were *c-MET* positive determined by histology H score of at least 150. Median PFS was 5.9 months (95% CI 2.8 to not reached). ORR was 30.6% (95% CI 16.3–48.1), and in those who were *c-MET* high defined as H score 225 or greater, ORR was 52%. The most common adverse events were neuropathies (57%) and dermatitis acneiform (38%), with grade 3 treatment-related AEs in 31% of patients, most frequently hypophosphatemia (7%) and peripheral sensory neuropathy (7%) [57].

### 5.4. EGFR-MET Bispecific Antibody

Amivantamab is a bispecific antibody for both EGFR and MET. Amivantamab was studied in patients with advanced NSCLC in cohorts based on *EGFR* and *MET* status [58]. In the CHRYSALIS phase 1 trial, amivantamab was studied alone or in combination lazertinib in patients with metastatic or unresectable NSCLC with *EGFR* mutations. Results have been presented at ASCO and ESMO. In patients who had progressed on osimertinib, an ORR of 36% was seen in those treated with combination amivantamab and lazertinib, with median PFS of 4.9 months. This is compared to ORR 19% and median PFS 4.2 months with amivantamab monotherapy [59,60]. CHRYSALIS 2 explored the combination of amivantamab and lazertinib in patients with *EGFR* mutant advanced NSCLC who had progressed after osimertinib and platinum-based chemotherapy. In this population, an ORR of 33% was seen [61]. 

In the CHRYSALIS trial, a cohort of patients with *EGFR exon 20* insertion mutations were also enrolled. In patients with locally advanced or metastatic NSCLC with *EGFR exon 20* insertion mutations who had progressed on or after platinum-based chemotherapy, an ORR of 40% (95% CI 29–51) was seen. Amivantamab received accelerated FDA approval in this setting based on these results [62].

In safety analyses of amivantamab, the most common adverse events were rash (86%), infusion-related reactions (66%), and paronychia (45%). Grade 3 or greater adverse events occurred in 35%, most commonly hypokalemia (5%), rash, pulmonary embolism, diarrhea, and neutropenia (all in 4% each) [58]. The MARIPOSA and MARIPOSA-2 phase III trials of amivantamab and lazertinib (third generation EGFR TKI) as first-line therapy for *EGFR*+ NSCLC are ongoing. 

## 6. Future Directions

There are several areas of clinical study that can help to move the field forward for *MET* amplification as a resistance mechanism to EGFR TKIs. Establishing a universal definition of *MET* amplification or overexpression with a focus on FISH and NGS as more sensitive diagnostic methods will be important in standardizing reporting establishing clinical trial eligibility. While tissue and cfDNA NGS have had significant uptake in recent years, it is unclear to what degree *MET* FISH is being performed for NSCLC off protocol and as standard of care. The optimal method for targeting *MET* amplification or overexpression has yet to be determined. As detailed above, MET is being targeted by TKIs, antibodies, and antibody drug conjugates. It is unclear if one particular drug or modality will have a clear advantage as far as efficacy or toxicity, or if these decisions will need to be made on an individual patient basis. Additionally, as clinical trials are now being designed to move *MET*-directed therapy to frontline treatment for EGFR-mutated NSCLC patients, it is yet unknown if this will yield benefit for all patients, or if additional biomarker studies are needed to understand exactly which patients benefit from a combination approach.

## 7. Conclusions

Identifying driver mutations and targeted therapies has developed a new standard of care for many patients with NSCLC. By identifying the genetic drivers of EGFR inhibitor resistance in a similar way of identifying initial driver mutations, the same principle of targeting these genetic drivers could be a strategy to delay progression and potentially delay the need for chemotherapy. In studies of genetic alterations after progression on osimertinib, *MET* dysregulation, particularly *MET* amplification, has been identified as a common mechanism of resistance. However, identifying a standardized definition of *MET* amplification for trial eligibility is needed. 

Multiple drugs that inhibit MET have been studied in advanced NSCLC, including tyrosine kinase inhibitors, antibodies, and antibody-drug conjugates. Combining EGFR inhibition and MET-directed therapy has been a promising area of clinical study. In early clinical trials, both combination TKI therapy, and combination TKI and antibody, antibody-drug-conjuate, and bi-specific EGFR-MET antibody have shown anti-tumor activity. Large-scale clinical trials of combination EGFR-MET inhibition are needed to understand the clinical benefit of this treatment strategy, as well as to consider this strategy as potential first-line therapy in advanced *EGFR* positive NSCLC. 

## Figures and Tables

**Table 1:** Trials targeting both EGFR and MET using EGFR and MET TKIs.

EGFR TKI	MET TKI	Study (NCT ID, Name, Author, Year)	Population	Treatment	MET Alteration	N	Objective Response Rate (ORR)	Progression Free Survival (PFS)
Gefitinib	Capmatinib	NCT01610336, Wu et al., 2018 [41]	*EGFR*+ NSCLC acquired resistance to first- or second-generation EGFR TKI	Gefitinib 250 mg once daily + Capmatinib 400 mg twice daily	IHC 3+, IHC 2+ plus *MET* GCN ≥ 5, or *MET* GCN ≥ 4	100	29%	5.5 months
Osimertinib	Savolitinib	NCT02143466, TATTON, Sequist et al., 2020 [42]	*EGFR*+ NSCLC progressed after prior therapy	Osimertinib 80 mg once daily + Savolitinib 300 mg once daily	IHC 3+, *MET* GCN ≥ 5 or *MET*/CEP ≥ 2:1	138	33–67%	5.5–11.0 months
			*EGFR+ T790M*- NSCLC with no prior third-generation EGFR TKI			42	62%	9.0 months
Osimertinib	Savolitinib	NCT03944772, ORCHARD, Yu et al., 2021 [43]	*EGFR*+ advanced NSCLC with progression on first-line osimertinib	Osimertinib 80 mg once daily + Savolitinib 300 or 600 mg once daily	*MET* amplification or exon 14 skipping by NGS	20	41%	Not reported
Gefitinib	Savolitinib	NCT02374645, Yang et al., 2021 [44]	*EGFR*+ advanced NSCLC progressed on prior EGFR-TKI with *MET* amplification	Gefitinib 250 mg once daily + Savolitinib 600 mg once daily	*MET* amplification by FISH GCN ≥ 5 or *MET*/CEP ≥ 2:1)	51	31%	4.0 months
Erlotinib	Capmatinib	NCT01911507, McCoach et al., 2021 [45]	Advanced *MET*-positive NSCLC (Cohort A *EGFR*+)	Erlotinib 100–150 mg once daily + Capmatinib 100–600 mg twice daily	FISH GCN or *MET*/CEP outside of normal range, IHC 2-3+, +RT-PCR, or exon14 splice mutation	12	50%	Not reported
Gefitinib	Tepotinib	NCT01982955, INSIGHT 1, Liam et al., 2023 [46]	Advanced/metastatic *EGFR*+ NSCLC acquired resistance to first- or second-generation EGFR TKI T790M-, no prior MET therapy	Gefitinib 250 mg once daily + Tepotinib 500 mg once daily vs. Chemo- therapy	IHC 2+ or 3+, *MET* GCN ≥ 5, or *MET*/CEP ≥ 2:1	55	45% (vs. 33% with chemo- therapy)	4.9 months (vs. 4.4 months with chemotherapy)
					*MET* amplification by FISH GCN ≥5 or *MET*/CEP ≥2:1)	19	68% (vs. 43% with chemo- therapy)	16.6 months (vs. 4.2 months with chemo-therapy)
Osimertinib	Tepotinib	NCT03940703, INSIGHT 2, Mazieres et al., 2022 [47]	Advanced *EGFR*+ NSCLC with *MET* amplification after progression on first-line osimertinib	Osimertinib 80 mg once daily + Tepotinib 500 mg once daily	*MET* amplification by FISH GCN ≥ 5 or *MET*/CEP ≥ 2:1)	22	55%	Not reported

EGFR: Epidermal growth factor receptor; MET: Mesenchymal-epithelial transition; TKI: Tyrosine kinase inhibitor.

**Table 2:** Trials targeting both EGFR and MET using either antibodies or antibody drug conjugates.

Mechanism Studied	Study (NCT ID, Name, Author, Year)	Population	Treatment	MET Alteration	N	Objective Response Rate (ORR)	Progression Free Survival (PFS)
EGFR TKI + MET antibody	NCT01900652, Camidge et al., 2022 [54]	Metastatic stage IV NSCLC with acquired resistance to erlotinib	Erlotinib 150 mg once daily + Emibetuzumab 750 mg 1.5-h infusion once every 2 weeks	IHC 2+	83	3%	2.9 months
EGFR/MET bispecific antibody +/− EGFR TKI	NCT02609776 CHRYSALIS, Bauml et al. [56] and Leighl et al. [55]	Metastatic or unresectable *EGFR* mutant NSCLC progressed on osimetinib	Amivantamab 1050 mg or 1400 mg (if >80kg) once a week in cycle 1, every 2 weeks following monotherapy	N/A	121	19%	4.2 months
			+ lazertinib 240 mg once daily		45	36%	4.9 months
EGFR TKI + MET ADC	NCT02099058, Park et al., 2021 [56]	Advanced *EGFR*+ NSCLC progressed on prior EGFR TKI	Erlotinib 150 mg once daily + Telisotuzumab vedotin 2.7 mg/kg IV once every 3 weeks	IHC H-score ≥ 150	28	32.1%	5.9 months

EGFR: Epidermal growth factor receptor; MET: Mesenchymal-epithelial transition; TKI: Tyrosine kinase inhibitor; ADC: Antibody drug conjugate.

## Data Availability

No new data were created or analyzed in this study. Data sharing is not applicable to this article.
